# Laughing in Laryngoscopy, and a New Mode of Applying Solutions to the Larynx

**Published:** 1866-02

**Authors:** H. A. Johnson

**Affiliations:** 611 Wabash Avenue; Chicago, Ill.


ARTICLE VII.

### LAUGHING IN LARYNGOSCOPY, AND A NEW MODE OF APPLYING SOLUTIONS TO THE LARYNX.

By H. A. JOHNSON, M.D., of Chicago, Ill.

In using the laryngoscope, every one, I presume, occasionally meets with a case in which there is extreme difficulty in getting a view of the glottis and vocal cords. After using all ordinary means, I have sometimes been able to succeed by requesting the patient to laugh.

The process of laughing, not well described in most works on physiology, consists in a forcible expulsion of air, interrupted by a rapid alternation of approximation and separation of the vocal cords. The glottis is, at the same time, elevated and the pharynx widely opened. The movements, it is true, are rapid, but do not prevent the observation of the color, form, and physiological action of the parts. This method, I believe, is new, and, in some cases, valuable.

In the application of solutions to the vocal cords and interior of the larynx, advantage may sometimes be taken of the laryngeal mirror to accomplish the result.

If a jet from the syringe be thrown directly in the line of vision upon the mirror while the structures below are in sight, the reflected spray will be carried directly down upon the parts observed.

If the jet be thrown at the commencement of inspiration, it will reach the interior of the larynx below the true cords. The result is an almost immediate closure of these cords, but I have been able to see the solution falling upon their upper surface, while the expired air passed up in bubbles between them. It is well, as in other cases, to request the patient to sound the note *ah!* during the process.

I do not suggest this mode as applicable in all cases. The brush, or even a direct jet from the curved nozzle of a syringe, may often be more desirable. By the brush, especially, the extent of the application may be limited to the diseased parts.

611 *Wabash Avenue, Jan.* 21*st*, 1866.

